# Online evolution of a phased array for ultrasonic imaging by a novel adaptive data acquisition method

**DOI:** 10.1038/s41598-024-59099-z

**Published:** 2024-04-12

**Authors:** Peter Lukacs, Theodosia Stratoudaki, Geo Davis, Anthony Gachagan

**Affiliations:** University of Strathclyde, Electronic and Electrical Engineering, Glasgow, G1 1XW UK

**Keywords:** Acoustics, Imaging techniques, Photoacoustics, Imaging and sensing, Electrical and electronic engineering

## Abstract

Ultrasonic imaging, using ultrasonic phased arrays, has an enormous impact in science, medicine and society and is a widely used modality in many application fields. The maximum amount of information which can be captured by an array is provided by the data acquisition method capturing the complete data set of signals from all possible combinations of ultrasonic generation and detection elements of a dense array. However, capturing this complete data set requires long data acquisition time, large number of array elements and transmit channels and produces a large volume of data. All these reasons make such data acquisition unfeasible due to the existing phased array technology or non-applicable to cases requiring fast measurement time. This paper introduces the concept of an adaptive data acquisition process, the Selective Matrix Capture (SMC), which can adapt, dynamically, to specific imaging requirements for efficient ultrasonic imaging. SMC is realised experimentally using Laser Induced Phased Arrays (LIPAs), that use lasers to generate and detect ultrasound. The flexibility and reconfigurability of LIPAs enable the evolution of the array configuration, on-the-fly. The SMC methodology consists of two stages: a stage for detecting and localising regions of interest, by means of iteratively synthesising a sparse array, and a second stage for array optimisation to the region of interest. The delay-and-sum is used as the imaging algorithm and the experimental results are compared to images produced using the complete generation-detection data set. It is shown that SMC, without *a priori* knowledge of the test sample, is able to achieve comparable results, while preforming $\sim$10 times faster data acquisition and achieving $\sim$ 10 times reduction in data size.

Ultrasound is a powerful and widely used imaging modality that allows examination or inspection of optically opaque systems and structures. It is relatively simple to use, cost effective and safe compared to alternative techniques (e.g. X-ray, MRI) and these characteristics have made it the most commonly used imaging modality worldwide for medical and industrial applications when it comes to imaging beyond what lies on the surface^1,2^. The impact of ultrasound imaging in our society has been made possible because of the technological advancements in ultrasonic equipment: transducer materials, electronics, signal generators and computational capabilities are at the heart of all ultrasonic imaging achievements^3^. Consequently, data acquisition and signal processing methods have been developed to conform with the available instrumentation capabilities, which are transducer-based ultrasonic phased arrays in their vast majority. These have a fixed number of elements, which have a fixed position and pitch on the array configuration, fixed operational frequency and corresponding bandwidth. As a consequence, the limitations of transducer phased arrays are currently defining our capabilities in ultrasonic imaging. The present paper proposes a new data acquisition methodology that goes beyond them.

Data acquisition methods for ultrasonic imaging can be broadly classified into methods where focusing is done during the signal generation and those where focusing is done during post-processing. One data acquisition method that belongs to the second group is to capture the complete data set of signals from all possible combinations of generation and detection elements of a dense array (i.e. an array that fulfils the Nyquist sampling criterion). This data acquisition method is known as the Full Matrix Capture (FMC) in non-destructive evaluation^4^ and synthetic aperture imaging in medical ultrasound^5^ . The resulting data set contains the maximum possible information that can be measured for a specific location of the array. This enables more accurate analysis of internal features through better resolving capabilities^4^ and more advanced methods such as Vector Total Focusing Method (VTFM)^6^ and scattering analysis^7^. However, this advantage of the FMC comes with certain restrictions with respect to the number and configuration of array elements, slow data acquisition rates and large data volumes. Examples where slow data acquisition limits the use of this method are dynamic processes such as industrial process monitoring or flow imaging in medical ultrasound^8^, where data acquisition must be faster than the changes in the system being imaged. Examples where the number of array elements and the data volume is important are 3D ultrasonic imaging, where large number of elements are needed and the computational time quickly becomes considerable for ultrasonic imaging of volumes encountered in practice. The cost for data storage and retrieval becomes an additional parameter to consider in such cases.

The most simple solution to address all the above mentioned concerns with the FMC is to introduce sparsity to the array, which will limit the number of signals captured to below the number required by the Nyquist criterion. Existing approaches include those that address sparsity based on: (a) transducer phased array design, e.g. non-periodic sparse arrays^9–11^, random arrays^12^, spiral arrays^13^ and Vernier arrays^10^ each with their respective advantages and disadvantages^14^ and (b) those that address it through signal processing, where a sparse data set is captured and then the FMC data set is reconstructed^15^. An example is to use Deep Learning to artificially produce the remaining data of the Full Matrix^16^. To achieve this, training is carried out utilising considerably large volumes of data. The construction and labelling of such data sets is challenging and expensive due to the computational resources and computational times required. New image processing algorithms have also been proposed that are able to suppress grating lobes, while imaging with sparse arrays, such as the Phase Coherence Imaging (PCI)^17,18^. Applying PCI requires the internal features to be point scatterers, that reflect ultrasound uniformly at all angles, limiting its application when various feature types are considered^19^. Another data acquisition approach that is worth mentioning here is Plane Wave Imaging (PWI). PWI does not use array element sparsity but reduces the number of acquired signals by generating plane waves during the signal generation, followed by focusing in the signal processing stage^20,21^. However utilising this approach limits analysis of individual contributions of generation and detection element pairs. This can restrict the usage of further post processing of the data, in addition to the delay-and-sum method, in order to enhance results, such as extracting scattering information used for feature characterisation^7^. The approach taken in Ref.^22^ addresses this limitation by reconstructing the FMC data set based on an inverse back-propagation operation on images from PWI captured data.

An overarching theme between the above listed methods, is the fact that they acquire information uniformly with no discrimination as to how much information can be achieved at the given sensor positions. However, in most cases a considerable portion of the sample may not be of interest, meaning that the presence of regions of interest is sparse but what is missing is information of their location. If one small region of interest is present within a large object, it is not effective to acquire information uniformly throughout the sample. In order to increase efficiency towards faster data acquisition and reduced data volumes, a new data acquisition strategy must be developed that can adapt the array design according to the geometry and material of the test target, the presence and location of internal features, the processing/work environment and the capabilities of the ultrasonic imaging system. This study proposes such a data acquisition strategy for phased arrays, the Selective Matrix Capture (SMC). In this approach the ultrasonic array adapts to the demands of the inspected target, on-the-fly. SMC requires a high degree of flexibility with respect to the various characteristics of the ultrasonic array sensor. Flexibility in array geometry, wide bandwidth, simultaneous excitation of several ultrasonic modes can be used to extract information and adapt the array design during data acquisition. This paper presents a first implementation of the SMC that focuses on adapting the array geometry and this degree of flexibility can be achieved using Laser Induced Phased Arrays (LIPAs)^23^.

LIPAs are synthetic arrays, based on the principles of laser ultrasonics (LU). Unlike transducer-based ultrasonic probes, LU is a completely non-contact method that can operate remotely, does not require any couplant and can adapt itself to complex geometries, addressing some current limitations of transducer-based ultrasonic arrays. In LIPAs, data acquisition is carried out by one generation and one detection laser, scanned independently of each other, allowing any arbitrary array design, with decoupled generation and detection layouts^11,23^.

The aim of this paper is to introduce the novel, adaptive acquisition strategy of SMC, wherein the array characteristics can be optimised and adapted to the needs of the specific ultrasonic imaging requirements. Knowledge of the inspected structure is built-up during the data acquisition process and the array configuration continuously evolves based on the latest acquired data. Thereby, the phased array characteristics evolve during ultrasonic imaging. This is achieved by a two-stage process, with the purpose of the first stage being to rapidly identify regions of interest, followed by the second stage, where the array parameters are optimised for accurate scatterer characterisation. The overall aim is to increase data acquisition efficiency with respect to data acquisition speed and data volume, while maintaining the high imaging quality provided by an FMC data set. Finally, a bespoke array is synthesised for each ultrasonic imaging case. This includes customisation of: element number, element location, array aperture, de-coupled generation and detection array element locations. This new capability means that the best ultrasonic array design is synthesised each time. The degree of freedom in ultrasonic array design offered by LIPAs is used as a tool to showcase the capability of the SMC in the imaging example presented in this paper.

The paper presents a first implementation of the SMC on a simple imaging case of a single scatterer using a LIPA for data acquisition. In this example, another novelty is presented which is a criterion based on the distribution of the intensity of pixels in the produced delay-and-sum image in order to stop the iterative process and proceed to array optimisation. Consequently, this data acquisition process lends itself well to automation towards higher ultrasonic imaging speeds and robotic implementation.

The SMC is demonstrated experimentally using LIPAs on an aluminium sample, with a single, omni-directional scatterer located at a depth of 15 mm. A comparison with the commonly used FMC acquisition, capturing data uniformly over the imaged region is presented to demonstrate the increase in acquisition speed and reduction of data size, allowed by selectively capturing only information-rich signals without *a priori* knowledge of the inspected structure. Two scenarios of array synthesis are considered for ultrasonic imaging, with respect to the location of the array versus the location of the scatterer. Scatterer located in: (a) a high array sensitivity region and (b) in a decreased array sensitivity region. These two scenarios imitate corresponding ultrasonic imaging situations. Furthermore, two schemes for array optimisation are developed and presented, following the scatterer detection stage.

## Methodology

**Figure 1:**
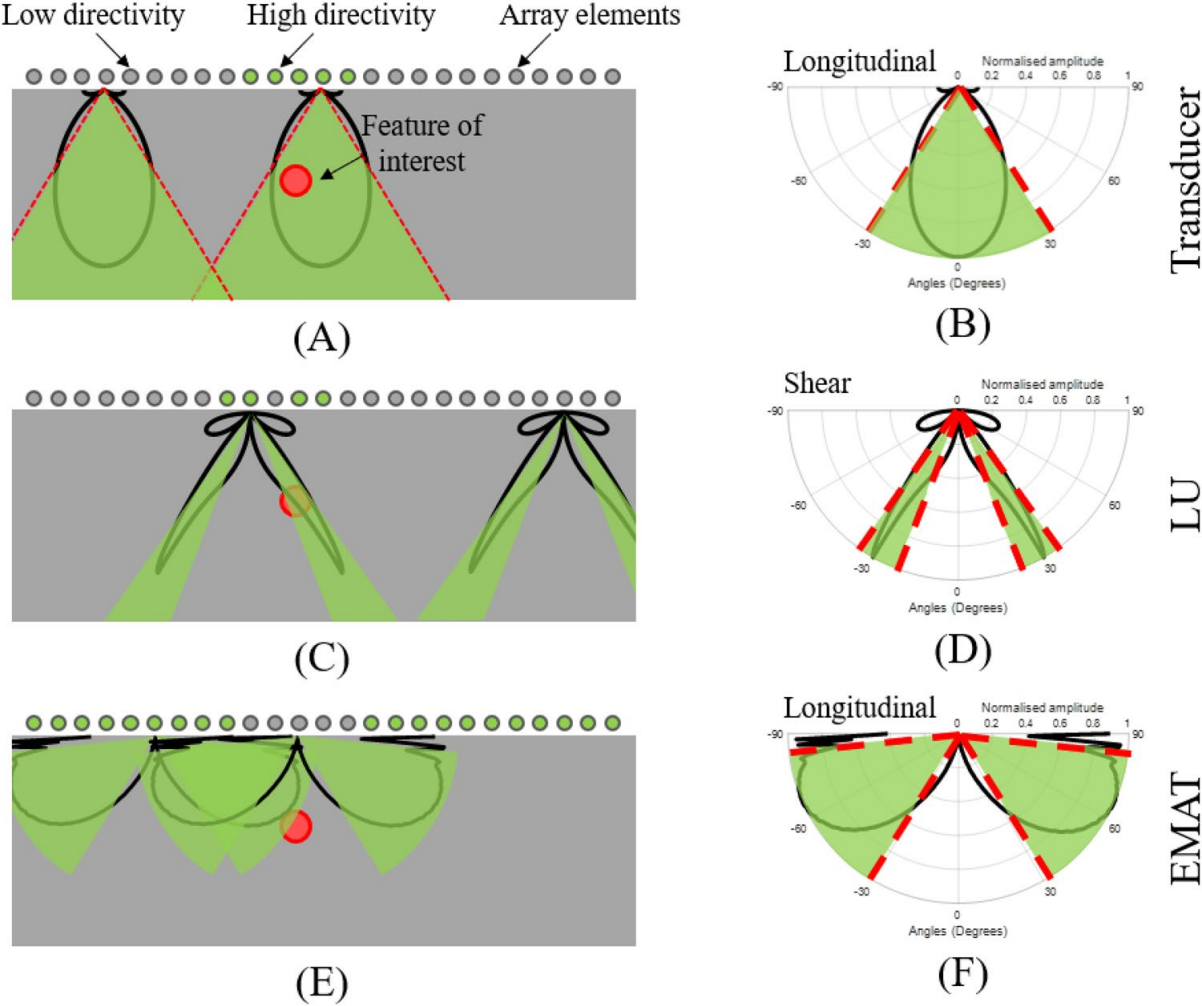
(**A**,**C**,**E**) Graphical demonstration of the amount of information contained in each signal, with respect to the location of the ROI for three array directivities: (**A**) Transducer array; (**C**) LIPA; (**E**) EMAT array. Red circle depicts ROI. Green and grey circles depict array elements with high or low directivity towards the ROI respectively. (**B**,**D**,**F**) directivity patterns for array elements from: (**B**) a transducer array; (**D**) a LIPA; (**F**) an EMAT array. Green areas highlight angles where directvity is higher than half relative to its maximum.

Array characteristics can affect the ultrasonic array sensitivity to scatterers depending on their location^24^. Thus, the location of a scatterer can influence which array elements will have the highest directivity and sensitivity for that specific position. The signals from these array elements will be information-rich, whereas signals from other elements will contribute mostly to noise. This is depicted in Fig. 1 for three different array directivity scenarios, corresponding to three different ultrasonic array methods: a transducer phased array, a LIPA and an Electro-Magnetic Acoustic Transducer (EMAT) array^25^. Figure 1B,D,F show example directivity patterns for: (a) the longitudinal wave mode of a transducer element^26^ ; (b) the shear wave mode of a laser generated ultrasound element^23^ and (c) the longitudinal wave mode of an EMAT element^27^. Figure 1A,C,E depicts the elements of the array that have high directivity towards the ROI (green circles) and will contain information-rich signals, while the elements that have low or no directivity towards the ROI (grey circles) will give signals that contribute mostly to noise in the ultrasonic image. The adaptive SMC method has the potential to be universally applied to phased arrays regardless of ultrasonic generation and detection methods, and their characteristics, such as wave mode, directivity and frequency content. In this paper LIPAs are used for the SMC implementation due to their high degree of flexibility towards the array design, as mentioned in the previous section.

**Figure 2:**
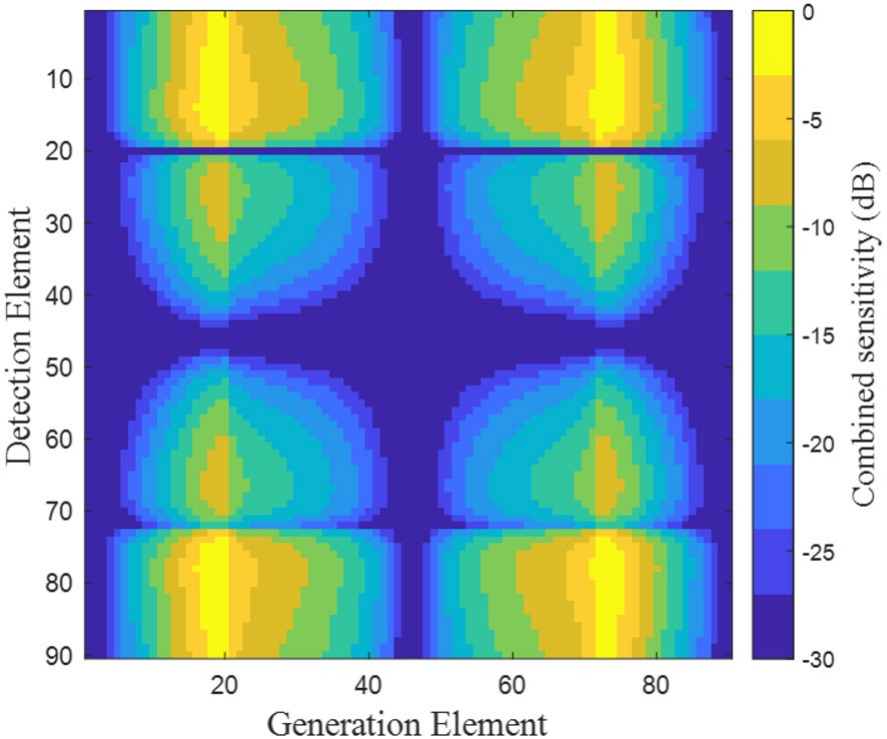
Matrix showing combined sensitivity of each generation and detection element combination for a point scatterer located at the centre of an array at a depth of 15 mm. The array consists of 90 elements and has an aperture of 30 mm.

Figure 2 shows the product of the laser ultrasound, shear wave mode generation directivity and detection sensitivity (termed combined sensitivity) for a scatterer located at a depth of 15 mm, at the centre of the array, for each element of an array combination. The array in this case consists of 90 elements, with an aperture width of 30 mm. This figure demonstrates that for this specific case, high combined sensitivity is achieved at around generation elements 20 and 70 and detection elements 10 and 80, while other elements will experience significantly lower combined sensitivity due to their relative location to the scatterer.

SMC exploits the concept demonstrated on the combined sensitivity in Fig. 2 through a two stage process. The initial stage involves a rapid scan with a sparse array of low element count and equidistant, large pitch, covering the entire available scan area as array aperture. The images generated are of low quality but provide enough information for detection and localisation of a ROI. Having obtained this information the second stage is performed, where an image with high quality is provided by an array optimised for the ROI identified during the first stage. For the second stage, two alternative configurations are proposed for an array focused and optimised for the ROI. The first array configuration has the array aperture segmented and limited around the location of highest directivity and sensitivity and array elements have equidistant pitch, satisfying the Nyquist limit ($\lambda$/2). The alternative design makes use of the entire available scan area as array aperture, utilising an array layout with varying element density based on the sensitivity to the scatterer. By considering the directivity and sensitivity patterns, more elements are placed in regions of high sensitivity, while keeping the number of elements low where sensitivity is lower. This may sound counter-intuitive at first, however the SMC approach followed in this paper follows the adaptive sensing concept where information -and consequently signal-to-noise ration (SNR)- is maximised to the (already identified) area of interest^28^. An alternative SMC approach can be an array element distribution for maximum angular information for the region imaged. Finally, if the first stage indicates no ROI in the test object the process ends, leading to a very fast data acquisition.

Background information related to laser ultrasonics, LIPA data acquisition and image processing and array sensitivity maps can be found in section 2 of the Supplementary Information. The following subsection is a detailed description of the proposed adaptive acquisition method.

### Selective matrix capture

#### Stage 1: detection and localisation of region of interest

The first stage of SMC is aimed at locating potential ROIs where a scatterer may be present. It is an iterative process where scatterer detection is performed after each iteration. An iteration involves capturing data with a defined pitch, while data processing is performed in parallel on the already captured data. At the end of each iteration, if the ROI cannot be located decisively, a new iteration is carried out with increased number of elements and smaller pitch. The iterations continue until the location of the scatterer can be decisively stated.

At this stage, it is expected that grating lobes will be produced, deteriorating imaging quality. This is due to the large pitch, periodic array that does not satisfy the Nyquist sampling criterion^26^. In order to reduce the negative effect of these grating lobes, Vector Coherence Factor (VCF) is utilised (see section 2 of Supplementary Information). Localisation of the ROI follows this imaging step.

There are several different scatterer detection processes that can be used to end the first stage. These include approaches based on setting an amplitude threshold^29^ or by using machine learning^30^. However the former assumes consistent sensitivity due to the unchanging array parameters of transducer-based arrays, as well as a robust calibration process, while the latter requires large volumes of data for training a neural network. A novel pixel distribution based method is proposed here. It is targeted towards imaging for non-destructive evaluation, and is suitable for this first demonstration of SMC described in this paper, as it is compatible with the reconfigurable array parameters of LIPAs and does not require large volumes of data for training. The method exploits the fact that noise in a delay-and-sum image will exhibit a certain pixel intensity distribution^31,32^. The nature of the noise appearing in ultrasonic images is complex, but can generally be divided into two categories: incoherent (such as electric/instrumentation noise) and coherent (such as inhomogeneity of ultrasonic propagation medium, undesired wave modes, artefacts produced by grating lobes, reflection form undesired features)^33^. If an image is considered that only contains noise (i.e. no signal from scatterer above the noise floor), then the distribution of pixel intensities will display a Rayleigh distribution. After normalising the image (see section 2 of the Supplementary Information) to the highest intensity pixel, the starting point of this distribution will appear at the highest intensity pixel bin (i.e. 0 dB). Note that the normalisation and conversion to dB scale will distort the curve, however a distribution, termed here as “characteristic distribution”, will be present (i.e. a Rayleigh curve converted to a logarithmic scale). When the imaged data contain a coherent signal above the noise distribution (as would be the case for detected waves from some form of internal scatterer), then pixels with intensity higher than the characteristic pixel intensity distribution observed for the noise floor, will be present in the delay-and-sum image (note that this is the characteristic distribution of the noise-floor and it is not the average values of the image). When the image is normalised to the amplitude of this scatterer, a shift of the distribution of the noise pixel values compared to a scatterer-free case will be observed. In an ideal case, when no artefacts are present, this shift will not occur. Thus scatterer detection and localisation can be carried out by observing the distribution of pixel intensities.

**Figure 3:**
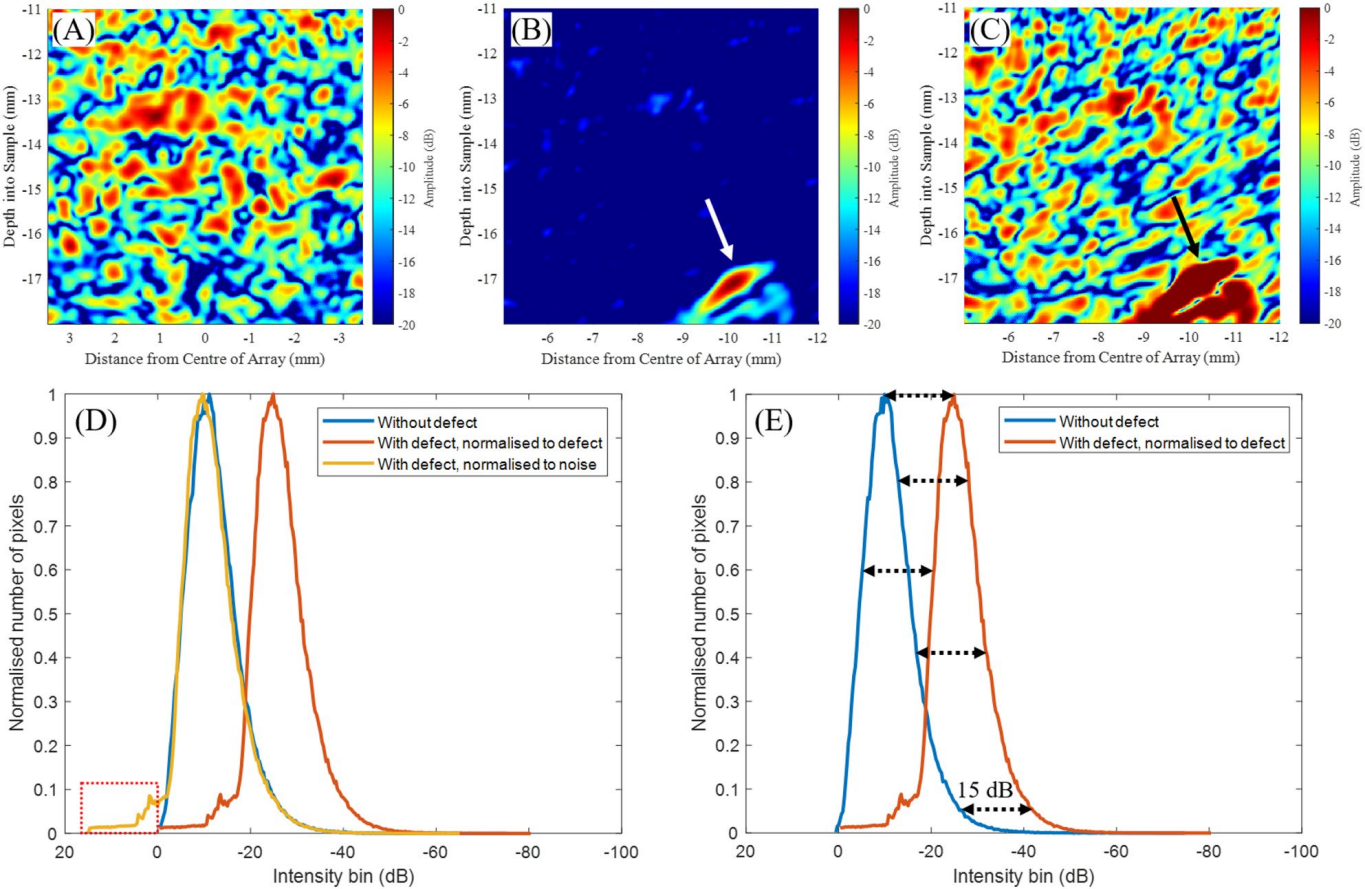
Ultrasonic delay-and-sum images, using experimental data from an array with 161 element and 0.155 mm pitch, of (**A**) a scatterer free region and (**B**,**C**) a region with a scatterer. White and black arrows show the location of the scatterer. Normalisation was performed on (**A**,**C**) the highest intensity noise pixel and (**B**) highest intensity scatterer pixel. (**D**) Graph shows the pixel intensity distribution of each ultrasonic image. Additional (**E**) graph highlights the 15 dB shift observed between the pixel intensity distributions with and without a scatterer.

Figure 3 demonstrates the proposed method on an example experimental data set. An ultrasonic delay-and-sum image of a region with and without a scatterer (1 mm diameter hole) can be seen on Fig. 3A and B respectively, normalised to their highest intensity pixel (i.e. (A) - a noise pixel, (B) - scatterer pixel). Figure 3 (C) shows the image from (B) but normalised to its highest amplitude noise pixel, thus now pixels corresponding to the scatterer have values higher than 0 dB and are saturated in this image.

Figure 3D shows the pixel intensity distributions of images (A–C). The distribution produced by the noise floor can be seen on the blue curve, as there is only noise on the corresponding image (Fig. 3A). This distribution can be also observed in the scatterer case, on the red and orange curves, with corresponding images Fig. 3B and C, however in these cases there are values above the noise floor and these appear as higher values on the distribution (i.e.: left of the noise curve) indicated as a red dashed rectangle on Fig. 3D. A good agreement can be seen of the noise floor distribution on the blue and orange curves, where in both cases normalisation was carried out on the noise floor. However when normalising to the scatterer (red curve on Fig. 3E), and Fig. 3B, the noise floor shifts, indicated on Fig. 3E. Thus the position of the Rayleigh distribution can indicate whether a scatterer is present or not.

The first stage has two potential outcomes: lack of scatterers is confirmed, or one or several ROIs are detected and located. In the case of the former, the acquisition ends after the rapid first stage, while in the case of the latter, the SMC moves on to its second stage and an optimised array is synthesised to capture a high quality image of the ROI.

#### Stage 2: scatterer characterisation

**Figure 4:**
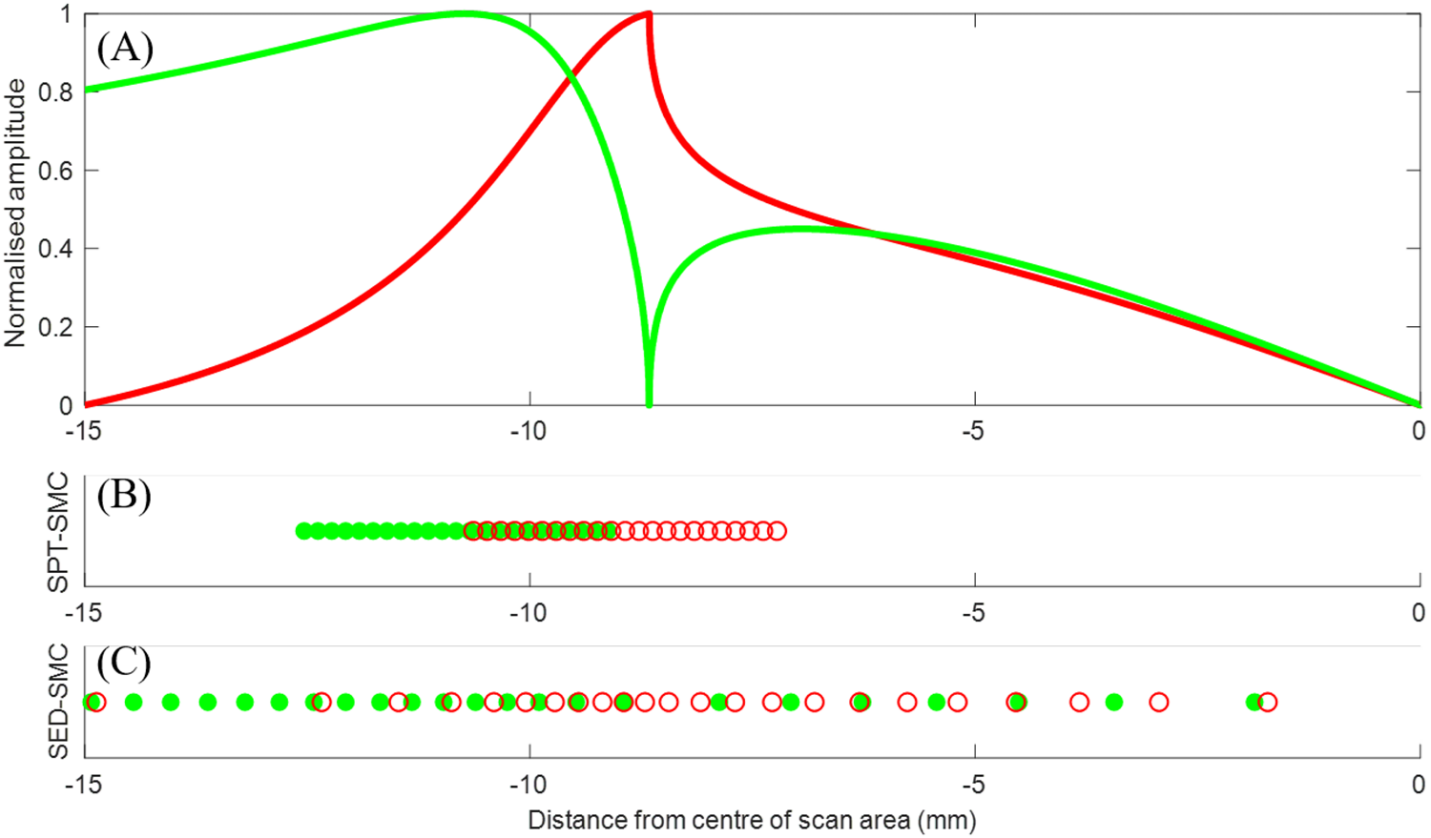
(**A**) Surface projected directivity (red curve) and sensitivity (green curve) patterns calculated for a scatterer located at 15 mm deep at 0 mm from the centre of the scan area. (**B**,**C**) Corresponding arrays produced using (**B**) SPT-SMC and (**C**) SED-SMC for the above shown patterns. Red circles and green dots are the generation and detection element positions, respectively. Figures are plotted for one half of the scan area (-15 to 0 mm) due to symmetry.

During the second stage, array element locations are selected based on the maximum achievable directivity and sensitivity for the ROI. For this task, the angular directivity and sensitivity patterns are first projected on to the surface for the specific ROI. These projections indicate the amplitude of the generated ultrasound and the sensitivity to an ultrasonic echo, specifically for the location of the ROI, as a function of potential generation and detection positions, as described by:

1 $$\begin{aligned} A_G(x_s)&= G_T\left( tan^{-1}\left( \frac{|x_s-x_P|}{z_P}\right) \right)&A_D(x_s)&= D_T\left( tan^{-1}\left( \frac{|x_s-x_P|}{z_P}\right) \right) \end{aligned}$$

where $x_s$ are the points along the surface of the sample where the surface projections ($A_G$, $A_D$) are calculated. $x_P$ and $z_P$ are the coordinates of the point defined by the centre of the ROI and $G_T$ and $D_T$ are the generation directivity and detection sensitivity (see section 1 of Supplementary Information), respectively. Figure 4A presents an example of a surface projected directivity (red curve) and a sensitivity (green curve). These were calculated for a scatterer placed at 15 mm deep at the centre of the scan area (i.e. 0 mm away from the centre).

The optimised array element positions are calculated for the ROI based on the surface projections, independently for generation and detection. Two methodologies are presented here for identifying the optimal array element positions, each presenting advantages and disadvantages: (a) the Surface Projection Threshold method (SPT) and (b) the Sensitivity-Based Element Distribution (SED). The former condenses the array elements within the regions, for optimal directivity and sensitivity, thus providing the highest possible SNR for a given number of array elements. However, confining the elements to small total aperture, restricts the angular aperture of the array, consequently restricting the maximum viewing angles measured. The latter method, SED, spreads the elements over a larger aperture, with varying element density based on the surface projected patterns. Unlike in the SPT approach, some elements are located in lower directivity and sensitivity regions hence, lower SNR is expected. However the larger aperture leads to increased lateral resolution. Furthermore, potential grating lobes produced by spreading the elements are suppressed due to the non-periodic layout of the array^34^. In both array designs, the increased element density in high sensitivity areas leads to delay-and-sum images with higher SNR at the ROI compared to that from an array with the same number of elements, equally spaced, within the same array aperture.

##### Surface projection threshold

A threshold is applied to the surface projected directivity and sensitivity in order to identify regions of high generation directivity and detection sensitivity. Elements are placed within these regions, using an equidistant, dense layout as shown on Fig. 4B. For representation purposes, only half of the surface projections are shown, as they display horizontal symmetry with respect to the ROI. The element locations of an example array designed using SPT can be seen on Fig. 4B).

LU exhibits narrow directivity and sensitivity patterns thus, the SPT optimisation process for array element positioning produces an array with small aperture, in general. This has a negative effect on the lateral resolution of the array. This resolution is dictated by the angular aperture, which is the range of angles that a point is insonified and viewed from.

##### Sensitivity-based element distribution

The second array optimisation utilises a non-linear element spacing based on the surface projected patterns. The array layout exhibits high element density and small pitch at regions with high generation and detection efficiency, while low density and large pitch for low sensitivity areas. In practice, array elements are located within the predefined array aperture, such that the integral of the surface projected directivity and sensitivity patterns between each pair of adjacent elements is equal to the integral of each other pair, according to the following equation:

2 $$\begin{aligned} \int \limits _{x_{n}}^{x_n+1} f(x) \ dx = \frac{\int \limits _{x_0}^{x_N} f(x) \ dx}{N} , \end{aligned}$$

where, x_0_ and x_N_ are the lower and upper limits of the predefined aperture, N is the number of array elements, *f*(*x*) is the function, in this case the surface projected patterns and $x_n$ is the position of the $n^{th}$ element, with $n=0,1,2 ... N-1$. An array produced using this sensitivity-based layout can be seen on Fig. 4C.

## Experimental configuration and test object

**Figure 5:**
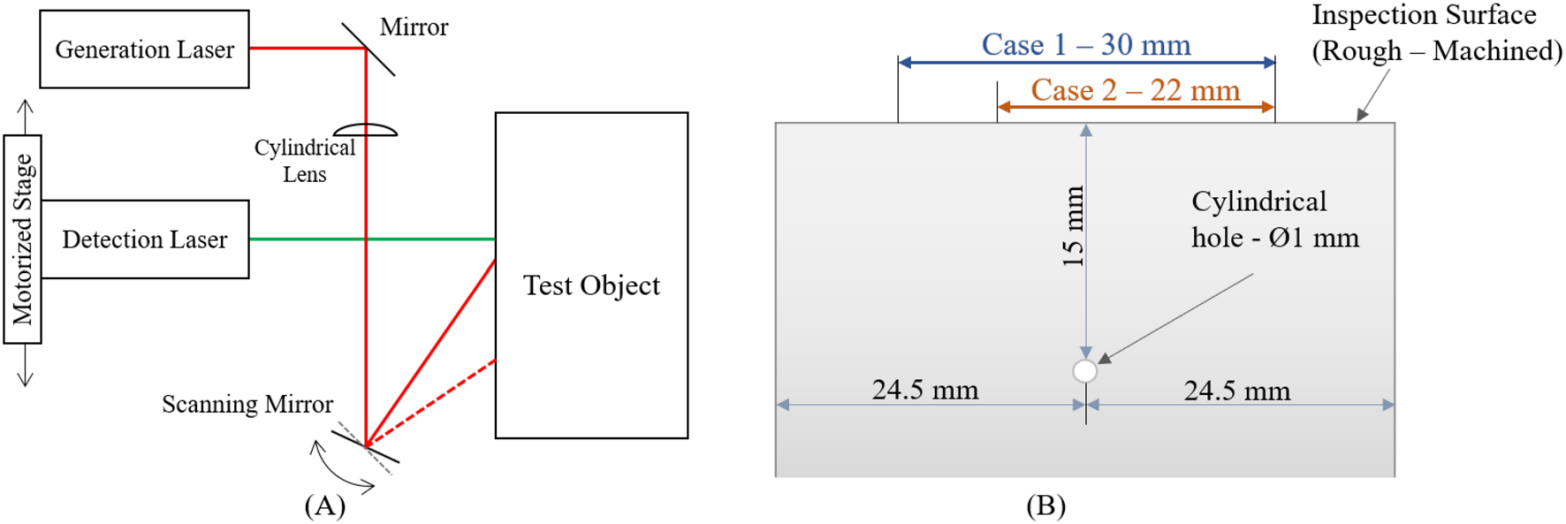
Diagram of (**A**) experimental setup and (**B**) experimental, aluminium sample. Cases 1 and 2 with their respective scan area of 30 mm and 22mm are indicated on the sample surface.

The experimental setup can be seen in Fig. 5. The laser used for ultrasound generation was a pulsed Nd:YAG, Q-switched laser (Elforlight, UK), with an optical wavelength of 1064 nm and a Full Width Half Maximum (FWHM) of 8 ns. This laser had 575 µJ energy per pulse and a repetition rate of 1 kHz. The beam was focused by a 200 mm focal length cylindrical lens to a line with dimensions of 0.56 mm x 3 mm. The generation laser was steered using a galvo-mirror (GVS302, Thorlabs) to achieve multiple generation lines on the surface.

The detection system used was a Quartet (Sound & Bright, USA) rough-surface interferometer. A continuous wave laser was used in the detector, with a wavelength of 532 nm and an average power of 780 mW. The detection system had a bandwidth of 1-66MHz and was sensitive to out-of-plane displacements. Each ultrasonic signal was averaged 32 times and captured using an oscilloscope (Agilent Technologies, InfiniiVision DSO5014A). The detection laser beam was incident normal on and scanned parallel to the surface of the test object. The scanning was facilitated using a motorised stage. The two lasers were scanned independently to synthesise the LIPAs.

The test object, with diagram shown on Fig. 5, was made of aluminium with a 1 mm diameter through-hole at the side. This cylindrical feature was chosen because it acts as an omni-directional scatterer, and will reflect ultrasound uniformly at all incidence and reflection angles. The through hole was created using Electrical Discharge Machining and it was located at a depth of 15 mm from the scanned surface. The sample surface had a rough, machined finish.

**Figure 6:**
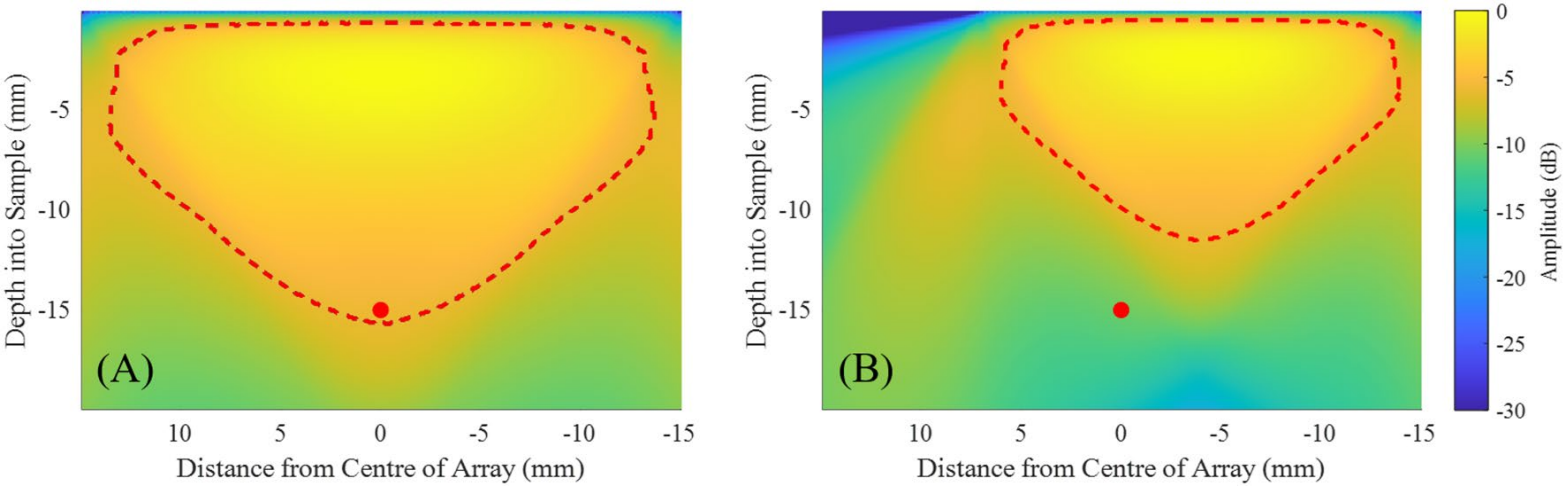
Array sensitivities for when the scatterer shown by a red circle is (**A**) inside and (**B**) outside the high sensitivity region between 0 and − 6 dB (red dashed region).

Two cases were considered for experiments (Case 1 and Case 2), with two different maximum imaging depths. This maximum depth is defined by the -6 dB region of the array sensitivities (see section 2 of the Supplementary Information). These calculations assume a linear equidistant array with a pitch of 0.155 mm, that satisfies the Nyquist limit up to 10 MHz. In Cases 1 and 2 the maximum imaging depths were selected to be 15.75 mm and 11.5 mm, respectively, with the array sensitivities shown on Fig. 6. The corresponding scan areas for Cases 1 and 2 were 30 mm and 22 mm, respectively. In addition, the scan area in Case 2 was offset such that the scatterer is 3.9 mm off-axis to the centre of the array, in order to intentionally reduce the available sensitivity (Fig. 6). Consequently, the scan areas are defined as the regions for possible element locations. Case 2 is presented in order to assess the performance of the SMC acquisition for a scatterer located outside the high sensitivity region and it is representative of the case when there is limited access for positioning the array to image the object.

## Results

In this section, the results are presented in two subsections, one for each stage of the SMC: stage 1, for detection of the region of interest and stage 2, for scatterer characterisation. In this example of SMC implementation presented here, the results from the proposed SMC method are compared to those from the acquisition that is commonly used for ultrasonic imaging for which equidistant, fully populated, dense arrays are synthesised within the region for possible element locations of Cases 1 and 2. Detailed description of these arrays is presented in subsection "Stage 2: scatterer characterisation".

The results presented in this paper were produced after applying filtering in post-processing to the experimentally captured data sets. The digital filter had a Gaussian shape and a 200$\%$ bandwidth with a centre frequency of 7 MHz. Images were produced by utilising the delay-and-sum algorithm and the VCF weighting (See section 2 of the Supplementary Information).

### Stage 1: detection and localisation of region of interest

Stage 1 of the SMC is an iterative process where progressively less sparse arrays are synthesised until a region of interest is detected, using the shift of the pixel intensity distribution produced by the noise in order to signal the end of this stage. As the shape, size and reflection amplitude of the scatterer is unknown, it is not possible to know the lowest number of elements required for defect localisation at the start of the data acquisition. Therefore applying such an iterative approach allows to perform the detection stage with as few elements as possible without requiring to populate a dense array, with many redundant elements.

If the peak of the distribution shifts beyond a predefined threshold relative to the peak produced by the first, very sparse iteration, then it signals the detection of the the ROI. For example, in the case presented, a − 6 dB threshold was empirically found to be sufficient. In the experimental cases described here, and for both Cases 1 and 2, three iterations were carried out, by the end of which the shift of the pixel intensity distribution relative to the characteristic distribution due to noise, was observed. Iterations 1 and 2 were necessary in case the scatterer manifested itself earlier, above the noise level, in which case this would signal the end of stage 1. Each iteration synthesised a LIPA with 10, 15 and 30 array elements. The array pitch were 3, 2 and 1 mm for Case 1 and 2.25, 1.5 and 0.75 mm for Case 2, utilising the entire region for possible element locations (30 and 22 mm for Case 1 and 2 respectively). The parameters of the arrays synthesised by Stages 1 of the SMC acquisition, including both cases 1 and 2, are presented in Table 1. Note that the data sizes are quoted for files saved in Matlab format.

**Figure 7:**
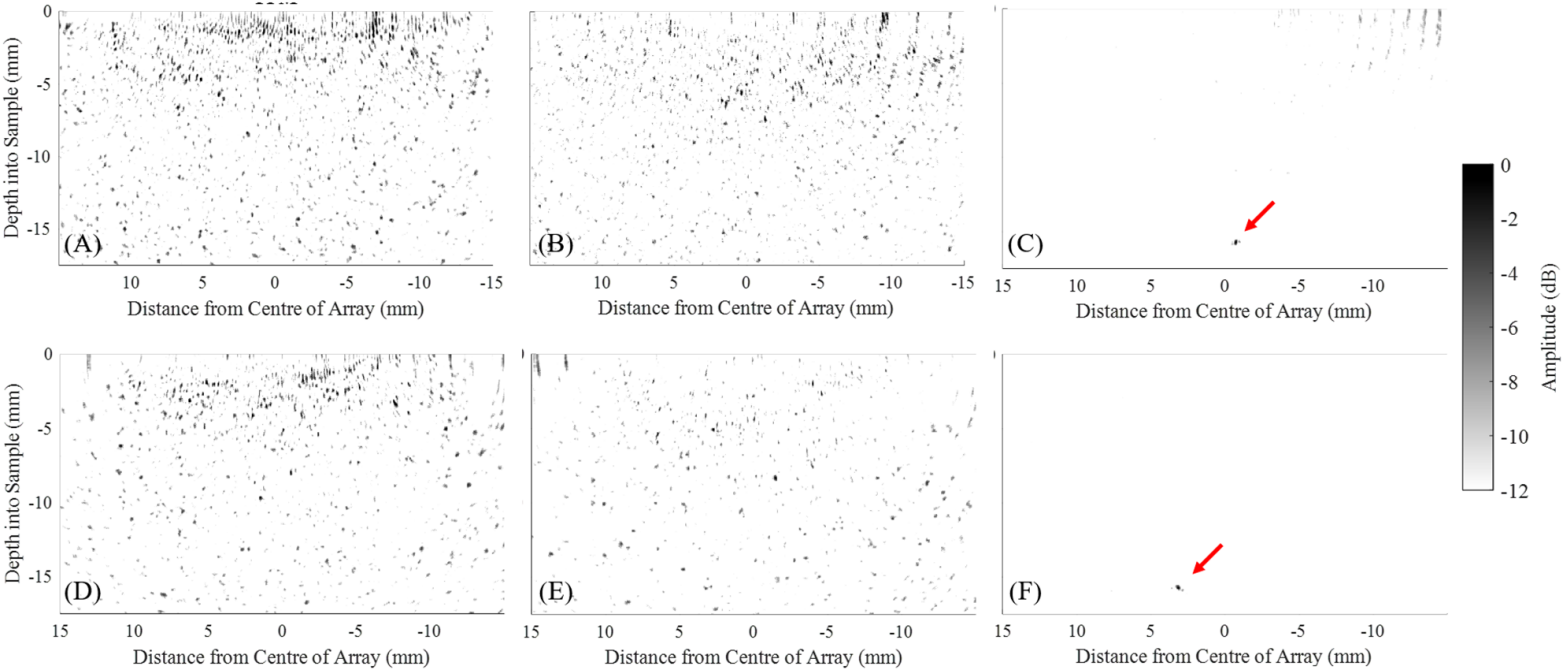
Experimental results from stage 1 of SMC for (**A**–**C**) Case 1 and (**D**–**F**) Case 2. In each case three iterations were performed: (**A**,**D**) iteration 1, (**B**,**E**) iteration 2 and (**C**,**F**) iteration 3. All images were plotted against the grey-scale dynamic range shown on the right. Red arrows indicate the location of the detect.

**Figure 8:**
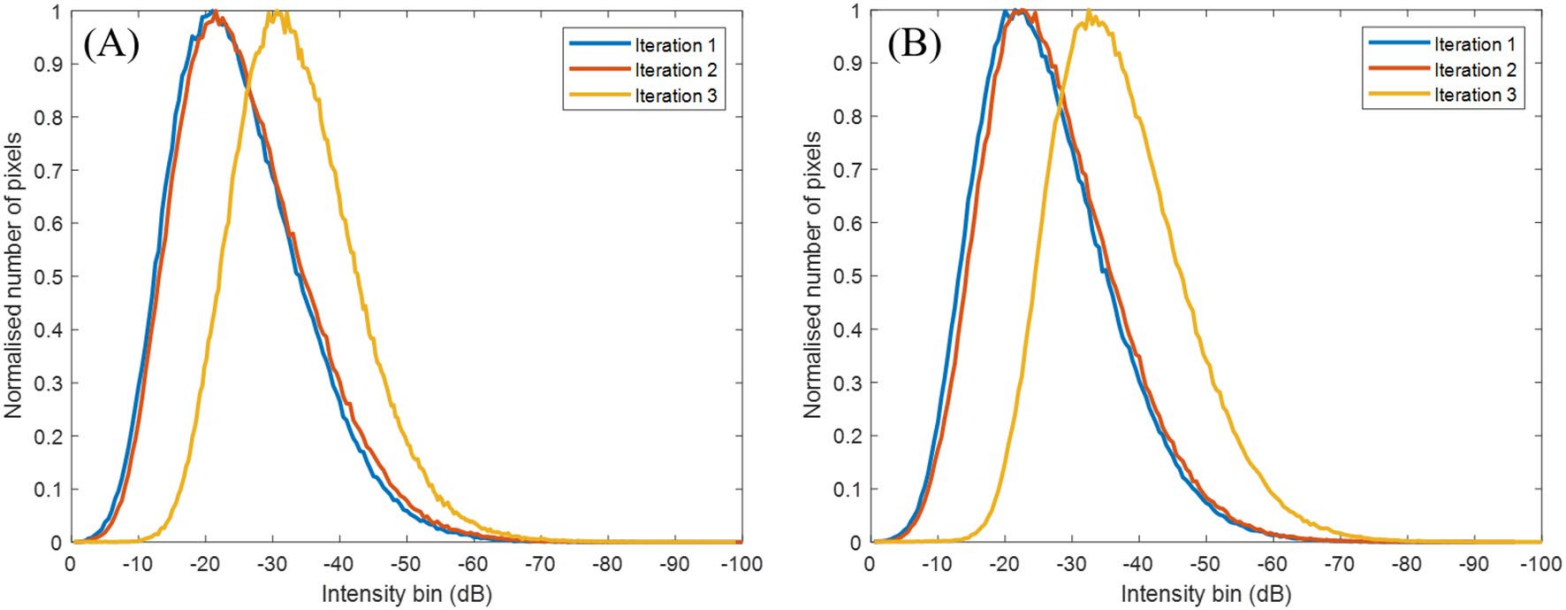
Pixel intensity distribution of images produced by the iterations of stage 1 shown for (**A**) Case 1 and (**B**) Case 2.

The acquired data sets were individually processed, producing a delay-and-sum image for each iteration. The 6 images produced can be seen on Fig. 7. The pixel intensity distribution of each delay-and-sum image was plotted and these are shown on Fig. 8A for Case 1 and (B) for Case 2. Pixels from the top surface of the sample (0 mm) to 5 mm deep were excluded in this analysis in order to exclude the artefact produced by the Surface Acoustic Wave (SAW)^23^. It is noted that SAW suppression was performed using the amplitude threshold method as described in Ref.^35^, however there was enough SAW residue left at the top section of the delay-and-sum image that it was affecting the analysis of the pixel intensity distribution. A clear shift of the characteristic distribution produced by the noise floor can be seen during the third iteration, for both cases. In Case 1, (Fig. 8A) the peaks of the curves for iterations 1 and 2 occur within 0.5 dB relative to each other, while the peak of iteration 3 is offset by 10 dB, relative to the average of the first two. For Case 2, the difference between the peaks of the curves of iterations 1 and 2 also occur within a 1 dB range relative to each other, while the peak of the third iteration is 11 dB lower than the average of the first two. The shift of the pixel values indicates that the scatterer’s intensity has increased above the noise floor during the third iteration, for both cases, and thus a ROI can be detected and located. This is visually validated by the images shown on Fig. 7, where the scatterer cannot be seen on (A–C) and (D–F), while it is visible on (C) and (F). The location of the pixels with values between 0 and − 12 dB are then defined as the ROI.

### Stage 2: scatterer characterisation

**Figure 9:**
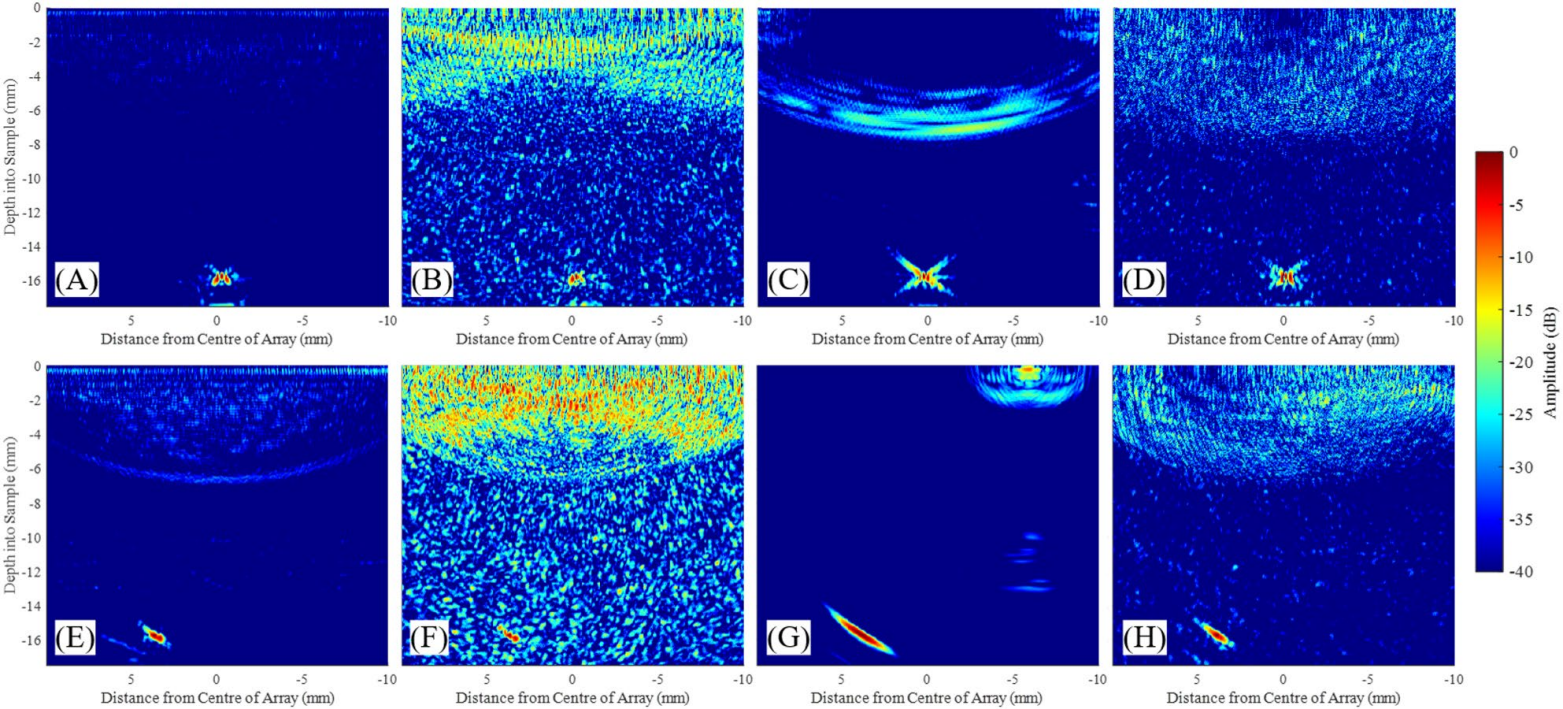
Ultrasonic delay-and-sum images produced for (**A**–**D**) Case 1 and (**E**–**H**) Case 2 by array configurations utilising: (**A**,**E**) FMC, (**B**,**F**) Sub-sampled FMC, (**C**,**G**) SPT-SMC and (**D**,**H**) SED-SMC. The dynamic range used in all images is indicated on the right.

During stage 1 the scatterer was located. The location of the scatterer was then used to design the optimised arrays. The location of the elements and the array distribution were based on the surface projected patterns as defined in subsections "Selective matrix capture". The captured data were post-processed using the delay-and-sum algorithm, and the results for Case 1 and Case 2 are shown in Fig. 9A–D and E–H respectively. In Case 1, both maxima of both the surface projected directivity and sensitivity patterns were within the possible array element location area, thus the array consisted of two sections for the SPT-SMC method, as presented on Fig. 4. The pitch of the array produced using this optimisation method was set to 0.155 mm, to satisfy the Nyquist limit, up to 10 MHz. Consequently, the array consisted of 46 elements. The alternative method for stage 2 array optimisation, the SED-SMC, was then designed to contain the same number of array elements as for SPT-SMC.

In Case 2, only one maximum of the surface projected patterns could be achieved, limited by the available scan area (i.e. 22 mm). In order to ensure that the effect of utilising only one maximum is decoupled from the effects of reduced sensitivity, the total number of elements for SPT-SMC, relative to Case 1, was reduced by the same amount that the scan region is reduced by (i.e. $\sim$27$\%$). This produced an array with 34 elements with a pitch of 0.103 mm, with its elements located in a single continuous aperture. The SED-SMC approach utilised 34 elements as well, distributed according to the surface projected patterns.

Figures 9A and E show the delay-and-sum images of the fully populated, FMC array, with its pitch satisfying the Nyquist criterion up to 10 MHz (i.e. 0.155 mm pitch) for Cases 1 and 2, respectively. Furthermore, a spatial down-sampling of this array was synthesised (Sub FMC in Table 1) for comparison of the proposed method to equally spaced arrays, with the same number of elements, in each case and results are shown on Fig. 9B and F. The aim for this comparison (between sub-sampled FMC and Stage 2 of SMC images) is to assess the efficiency and ultimately the quality of information contained in each captured signal, which is utilised towards the images produced, with (2nd stage SMC) and without (sub-sampled FMC) optimisation of the array based on the knowledge of the scatterer’s location. This sparse array had a pitch of 0.65 mm, the same aperture size as the array that provided the FMC data set and the same number of elements as the optimised array synthesised during the second stage of the SMC. The array parameters, corresponding experimental data acquisition times and the measured SNR are shown on Table 1. Note that all values quoted for SPT-SMC and SED-SMC in this table include the data captured during both Stages 1 and 2. Figure 9C,D,G,H were produced using data from Stage 2 only.

**Table 1:** Comparison of acquisition methods.

		Array elements	Number of signals	Pitch (mm)	Scan time	SNR (dB)	Data size (MB)
Case 1	FMC	193	37249	0.155	42 min	65.92	97.39
	Sub FMC	46	2116	0.650	2.5 min	40.46	5.62
	Stage 1	10/15/30	1225	3/2/1	1.5 min	–	3.29
	SPT-SMC	46	2116	0.155	2.5 min	65.93	5.60
	SED-SMC	46	2116	Varying	2.5 min	50.44	5.70
	Total SMC	–	3341	–	4 min	–	SPT: 8.89, SED: 8.99
Case 2	FMC	142	20164	0.155	23 min	63.72	55.83
	Sub FMC	34	1156	0.650	77 sec	36.52	3.23
	Stage 1	10/15/30	1225	2.25/1.5/0.75	82 sec	–	3.24
	SPT-SMC	34	1156	0.103	77 sec	69.96	3.18
	SED-SMC	34	1156	Varying	77 sec	52.28	3.10
	Total SMC	–	2381	–	2.65 min	-	SPT: 6.42, SED: 6.34

## Discussion

**Figure 10:**
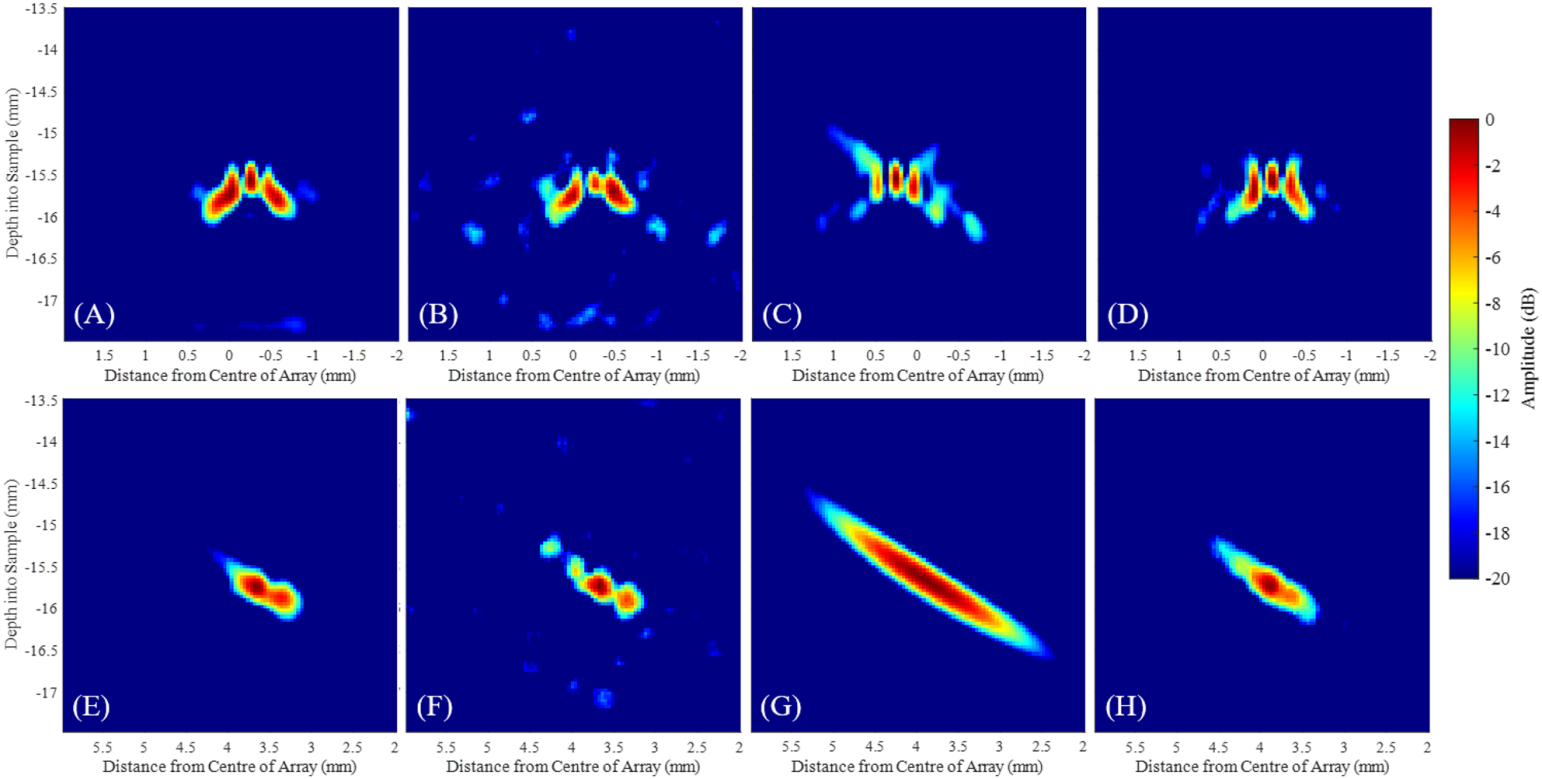
Close-up ultrasonic delay-and-sum images of the scatterer, produced for (**A**–**D**) Case 1 and (**E**,**F**) Case 2 by array configurations utilising: (**A**,**E**) FMC, (**B**,**F**) Sub-sampled FMC, (**C**,**G**) SPT-SMC and (**D**,**H**) SED-SMC.The dynamic range used in all images is indicated on the right.

A first implementation of the SMC data acquisition method, with its two alternative optimisation methods, SPT-SMC and SED-SMC, was demonstrated for the simple case of imaging a single scatterer located within an aluminium sample considering two cases with varying scan area sizes. In Case 1, during the second stage, the optimised array design using the SPT-SMC produced a delay-and-sum image of SNR equal to the delay-and-sum image using FMC and in Case 2, a delay-and-sum image of $\sim$6 dB higher SNR than the FMC array, utilising $\sim$17.5 times fewer ultrasonic signals, as shown in Table 1. Figure 10 presents close-ups of the scatterer from the images shown on Fig. 9. Figure 10C and G show a decrease in scatterer resolution, especially for Case 2, when only one sub-aperture was utilised. This can be explained by the reduced aperture size when compared to the FMC acquisition. In comparison, the SED-SMC array optimisation method produced images (Fig. 10D and H) with similar scatterer resolving capabilities to that of the FMC acquisition, as the entire available scan region was used for the array aperture, thus this array had a wider range of viewing angles. However, the overall SNR of the SED-SMC array was decreased compared to the image produced by SPT-SMC, as fewer elements were located at the peaks of the surface projected patterns in the SED-SMC array optimisation. Compared to the delay-and-sum image using FMC acquisition, a decrease of $\sim$ 15 and 11 dB were observed for Cases 1 and 2 when using SED-SMC.

The results from this SMC implementation example demonstrate that delay-and-sum images using data acquired by the SMC method achieved either comparable SNR (SPT-SMC) or comparable scatterer resolving capabilities (SED-SMC), while the experimental data acquisition time was $\sim$ 11 and $\sim$ 9 times faster than the FMC method, for Cases 1 and 2 respectively. SMC could enable the use of advanced processing algorithms such as VTFM, and scattering matrices, with significantly improved acquisition speed by only acquiring the sections of the Full Matrix that contain information. Furthermore, reduction of data size similar to the acquisition speed improvement was also observed. The data sets produced by the SPT-SMC and SED-SMC, including all signals from stage 1, were $\sim$ 11 and $\sim$ 9 times smaller than that of the FMC acquisition for Cases 1 and 2 respectively.

The optimisation in this work was carried out for a specific ROI location, obtained during stage 1 of the SMC. Three iterations were carried out, both for Cases 1 and 2 and the presence of a ROI could be identified based on the shift of the pixel intensity distribution relative to the characteristic distribution due to noise. The ROI can be easily located on the third iteration for both cases from the delay-and-sum images shown on Fig. 7. Analysing the distribution of pixel values of iterations 1-3, for Cases 1 and 2, a clear shift can be seen of the curve produced by the noise floor at iteration 3, by around 11 dB for both cases. This is in good agreement with what was observed on the delay-and-sum images, demonstrating the proof-of-concept of this method. In the future, this approach could be part of an automated SMC data acquisition system. In this case, scatterer detection can be improved by utilising a threshold of the pixel distributions. For example, a 6 dB threshold was sufficient for detecting the scatterer in the experimental cases described in this paper. However, the results of this example of a single scatterer cannot be generalised to all possible application scenarios of SMC and further studies should be carried out to establish a reliable and robust value for this threshold, considering other scenarios such as multiple scatterers and scattering properties.

In this work, the array designs presented during stage 2 of SMC were optimised for an omni-directional scatterer. If the scatterer exhibits a scattering directivity, which is preferential to the elements at locations that have no or low sensitivity (i.e.: elements not included in the stage 2 of SMC), then minimal information is acquired even if we capture signals at those combinations. Therefore, this is an ultrasonic phased array sensitivity problem and not a limitation of the proposed acquisition method. In such case, the ultrasonic imaging using the FMC acquisition method would suffer from the same level of degradation as the optimised stage 2 SMC array when compared to imaging an omni-directional scatterer.

Furthermore, the noise distribution methodology is designed to signal the presence of a ROI and it is not able to declare a region scatterer free, if this is indeed the case. It is critical for future implementations to consider a criterion to stop the iterative process of stage 1, when the sample is free of ROIs, before reaching the synthesis of a fully populated, dense array. This could be addressed by considering the probability of detection, which describes the capability of a system to detect scatterers as a function of true and false positive indications and is defined as the fraction of signals from scatterers that yield detectable indications^36^. The ultrasonic imaging application requirements would determine the sensitivity that should be attained using the minimum number of elements, while the probability of detection is still sufficiently high for the detection of the minimum expected scatterer size and maximum depth.

In the realisation of the SMC presented in this paper, highly sparse equidistant linear LIPAs were utilised during stage 1, the scatterer detection stage, however other sparse LIPA designs have been demonstrated (e.g. random arrays, Vernier) that are able to increase the array imaging ability with reduced number of array elements^11^. These designs could lead to the scatterer detection stage being able to locate the ROI earlier, further improving the speed of this method. These array designs are possible due to the flexibility of the Laser Ultrasound method combined with the adaptability of the SMC.

In the experimental example of the SMC methodology presented here, the SMC was demonstrated to significantly reduce the required acquisition times and data volume associated with cross-sectional ultrasonic imaging using linear arrays. There is an increasing need for 3D ultrasonic imaging in application for various fields, due to its ability to provide a better representation of features, in a 3D space. 2D arrays can be utilised to perform 3D imaging, however in order to satisfy the Nyquist limit for these arrays, with a sufficiently large aperture, considerably higher number of array elements are required than the case of 1D arrays. This demand hinders the application of 2D arrays due to the costly instrumentation (e.g.: probes, controllers) in the case of transducers, and the long acquisition times for LIPAs^37^. The SMC methodology could be applied for data acquisition of 2D arrays such as 2D LIPAs for 3D imaging, addressing these limitations of 2D arrays and this remains to be experimentally demonstrated.

Synthetic phased arrays using laser ultrasound are increasingly being used in non-destructive evaluation^38,39^ and medical ultrasound^40,41^. The majority of these researchers use some type of synthetic aperture method^9,42^ but do not capture the complete data set of signals from all possible combinations of ultrasonic generation and detection elements (FMC), despite the clear advantages for high resolution imaging^4,5^. This is because of the lengthy data acquisition process in laser ultrasonics, mainly due to lack of instrumentation for parallel signal detection. Researchers in laser ultrasonics that use FMC as data acquisition method are few^43–45^. The concept of SMC, where the array element positions are optimised during data acquisition and aiming at data acquisition efficiency, has the potential to increase the use of laser ultrasound for high resolution imaging. However, to realise this potential, other types of implementations of SMC, different from the one presented in this paper, should be developed to address cases such as, multiple scatterers, scatterers with a variety of scattering properties or diverse echogenisity of the target region. In this respect, the concept of SMC presents itself as an opportunity for novel research in the field of laser ultrasonics.

## Conclusion

This paper presents an adaptive acquisition strategy, named Selective Matrix Capture, which optimises the ultrasonic array parameters to the needs of the imaging situation, on-the-fly. The array optimisation was experimentally demonstrated with respect to the location of an internal feature of the test sample, resulting in an increase in data acquisition speed and reduction of data volume, while producing high quality images, conventionally afforded by an FMC data set. The process is performed with no *a priori* knowledge of the location of the internal feature, and information is built-up during the rapid initial stage of the data acquisition method, aimed at locating regions of interest. This is followed by a second stage where the array parameters are optimised for characterising the located ROI. During acquisition, the array configuration is continuously evolving according to the latest acquired data. SMC was achieved by utilising LIPA, a highly flexible, synthetic ultrasonic array, based on principles of laser ultrasonics, allowing for wide bandwidth and re-configurable array geometries with decoupled generation and detection layouts, including the ability to overlap elements.

In the first implementation of SMC presented here for the case of a single, high ultrasonic contrast, omni-directional scatterer, the SMC method achieved a tenfold improvement in data acquisition speed on average, when compared with the FMC acquisition method. In addition, LIPA is a remote, couplant free, optical based technique, which lends itself well to automation. Large area ultrasonic imaging using the LIPA implementation of SMC comes as a natural future development.

The concept of SMC, where optimisation of sensor positions is done during data acquisition in order to increase acquisition efficiency has a certain degree of universality and could be implemented using other types of ultrasonic sensors, such as transducer-based phased arrays or electromagnetic acoustic transducers. For example this concept could be applied to sensor positioning for robotic ultrasonic guided-wave grid mapping^46^. SMC could contribute to the ability of networks of multiple sensors to self-reconfigure for optimum data acquisition. For example, it would be interesting to see if the concept can be applied within the framework of swarm robots for non-destructive evaluation or Structural Health Monitoring, in order to enhance the autonomy and efficiency of such mobile robotic system^47^.

Finally, SMC is a methodology with data economy embedded in its concept. The data reduction achieved by the SMC is important in many respects: firstly because the data have been selectively captured to include information-rich data instead of mostly noise; secondly the reduced data size facilitates data storage and enables faster data processing allowing implementation of the technique in cases such as imaging of large objects, 3D imaging or capturing multiple frames of the same volume; and thirdly because the task of data transfer is facilitated.

### Supplementary Information

Supplementary Information.

## Data Availability

The data supporting this study are openly available from the University of Strathclyde Knowledge Base at: https://doi.org/10.15129/0950371c-0dc5-4eea-a7c9-007c135290a5.
