# Supplementary material for: Online evolution of a phased array for ultrasonic imaging by a novel adaptive data acquisition method
Source: Sci Rep. 2024 Apr 12;14:8541. doi: 10.1038/s41598-024-59099-z (PMC11015044; doi:10.1038/s41598-024-59099-z)
Supplement: Supplementary file 1 — Supplementary Information. [file 41598_2024_59099_MOESM1_ESM.pdf]

Supplementary Information

Online evolution of a phased array for ultrasonic imaging by a novel adaptive data acquisition method

Peter Lukacs, Theodosia Stratoudaki, Geo Davis, Anthony Gachagan
University of Strathclyde, Electronic & Electrical Engineering, Glasgow, G1 1XW, UK

1 Laser Ultrasound Generation and Detection

In laser ultrasonics, acoustic waves are generated and detected using lasers. Laser generated ultrasound produces all wave modes, including Surface Acoustic Waves (SAW), transverse and longitudinal bulk waves [1]. For the generation of ultrasound waves non-destructively, a thin volume on the surface of the test material is heated up by the absorption of the optical beam. The change in temperature then causes the material to expand thus generating ultrasound waves by inducing stresses and strains. For many materials such as metals, the absorption of laser light takes place within a depth of a few nanometers while thermal diffusion further extends the heated region to a depth of around one micron [2, 3]. As a result, the heating causes thermal expansion at times comparable to the rise time of the laser pulse (commonly of 1-10 ns duration) [4] and the induced ultrasound waves exhibit a wide-band spectrum.

Laser generates transverse waves more efficiently than longitudinal waves in this thermoelastic, non-destructive regime for the case of metals [5]. For example, shear waves induced in aluminium have amplitudes an order of magnitude larger than longitudinal waves [4].

The directivity patterns of laser generated transverse waves in the thermoelastic regime is defined by

$$G_T(\theta) = \frac{sin(2\theta)cos(2\theta)}{cos(2\theta)^2 + 2sin(\theta)sin(2\theta)(\kappa^{-2} - sin(\theta)^2)^{1/2}}, \tag{1}$$

where θ is the angle relative to surface normal and κ is the ratio of longitudinal to shear wave velocities. Similarly, the sensitivity of an out-of-plane laser detector, can be defined by

$$D_T(\theta) = \frac{sin2\theta(\kappa^2 sin^2(\theta) - 1)^{1/2}}{F_0(\kappa sin\theta)}. \tag{2}$$

Based on these equations, the resultant directivity and sensitivity patterns for a transverse wave can be viewed on fig. S1 (A) generation and fig. S1 (B) out-of plane component detection, using the material properties of aluminium.

2 Laser Induced Phased Arrays and Ultrasonic Sensitivity Maps

Laser Induced Phased Arrays utilise two lasers, one for ultrasound generation and another for detection to create a synthetic array of elements. The two lasers are scanned independently of each other, to every element position of the array, capturing a signal for each generation and detection element position combination [6]. Images are created in post-processing using the delay-and-sum (DAS) algorithm. In the DAS algorithm the ultrasonic signals are time-shifted to achieve dynamic focusing on every point (x, z) of a predefined grid [7] and can be defined by the following equation:

$$I(x, z) = \left| \sum_{tx=1}^{n} \sum_{rx=1}^{n} S_{tx,rx}(\tau_{tx,rx}(x, z)) \right|, \tag{3}$$

where n is the number of array elements, $S_{tx,rx}$ is the ultrasonic signal captured by tx and rx generation and detection element combination and $\tau_{tx,rx}(x, z)$ is the time delay defined by:

$$\tau_{tx,rx}(x, z) = \frac{\sqrt{(x_{tx} - x)^2 + z^2}}{c} + \frac{\sqrt{(x_{rx} - x)^2 + z^2}}{c}, \tag{4}$$

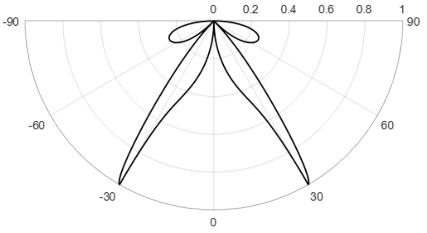

(A)

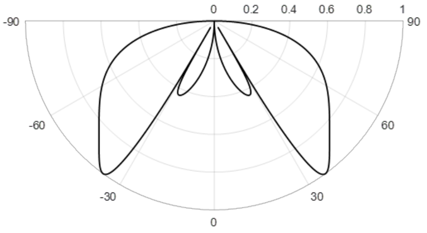

(B)

Figure S 1: Polar plots using normalised amplitude, (A) directivity pattern of laser generated ultrasound and (B) sensitivity pattern of an out-of-plane component laser ultrasound detector in aluminium, in the thermoelastic regime for transverse wave mode.

where x_{tx} and x_{rx} are the position of the generation and detection elements respectively and c is the acoustic velocity in the target material.
Furthermore, adaptive beam-forming can be utilised in order to enhance ultrasonic images by suppressing the grating lobes of sparse arrays [8]. In this work this is done by using the Vector Coherence Factor (VCF) due to its ease-of-implementation and increased performance compared to other phase coherence factors [9]. VCF is calculated by

$$V(x,z) = \sqrt{\left(\sum_{tx=1}^{n}\sum_{rx=1}^{n}\frac{Re(S_{tx,rx}(\tau_{tx,rx}(x,z)))}{|S_{tx,rx}(\tau_{tx,rx}(x,z))|}\right)^2 + \left(\sum_{tx=1}^{n}\sum_{rx=1}^{n}\frac{Im(S_{tx,rx}(\tau_{tx,rx}(x,z)))}{|S_{tx,rx}(\tau_{tx,rx}(x,z))|}\right)^2}, \quad (5)$$

where $S_{tx,rx}$ is the combination of the real and the imaginary parts of the detected signals. VCF is utilised as a weighting factor to produce the final image as described by

$$H(x,z) = I(x,z)V(x,z). \quad (6)$$

The images are normalised and converted to dB scale using

$$H_{norm}(x,z) = 20 \times log_{10}\left(\frac{H(x,z)}{max(H(x,z))}\right). \quad (7)$$

The delay-and-sum images produced will have varying sensitivity depending on the region imaged relative to the array and the characteristics of the array [10]. Thus the array is more sensitive to scatterers in certain imaging regions over others. This can be quantitatively defined by ultrasonic sensitivity maps [11], indicating the sensitivity of the array to an ideal point scatterer at any given pixel of the image. The directivity and sensitivity patterns, defined by eq 1 and 2, are used to calculate the amount of ultrasound wave propagating towards the given pixel and the detected portion of the reflected wave depending on the transmitted and received angles. Furthermore, beam-spreading caused by geometric attenuation is also accounted for. For a cylindrical wave the beam-spreading has an inverse square root relationship to the distance travelled by the ultrasonic waves. The sensitivity map can be described by the following equation:

$$E(x,z) = \sum_{i=1}^{n}\sum_{j=1}^{n}\frac{G_T(\theta_i(x,z))D_T(\theta_j(x,z))}{\sqrt{(d_i(x,z)d_j(x,z))}}, \quad (8)$$

where θ_i and θ_j are the angles to the pixel relative to generation and detection elements, d_i and d_j are the distances between the pixel and the generation and detection elements, respectively.

References

1. Scruby, C. B. & Drain, L. E. *Laser ultrasonics: techniques and applications* (Routledge, 2019).
2. Doyle, P. On epicentral waveforms for laser-generated ultrasound. *Journal of Physics D: Applied Physics* **19,** 1613 (1986).
3. Telschow, K. & Conant, R. Optical and thermal parameter effects on laser-generated ultrasound. *The Journal of the Acoustical Society of America* **88,** 1494–1502 (1990).
4. Rose, L. Point-source representation for laser-generated ultrasound. *The Journal of the Acoustical Society of America* **75,** 723–732 (1984).

5. Davies, S., Edwards, C., Taylor, G. & Palmer, S. B. Laser-generated ultrasound: its properties, mechanisms and multifarious applications. *Journal of Physics D: Applied Physics* **26,** 329 (1993).
6. Stratoudaki, T., Clark, M. & Wilcox, P. D. Laser induced ultrasonic phased array using full matrix capture data acquisition and total focusing method. *Optics express* **24,** 21921–21938 (2016).
7. Holmes, C., Drinkwater, B. W. & Wilcox, P. D. Post-processing of the full matrix of ultrasonic transmit–receive array data for non-destructive evaluation. *NDT & E International* **38,** 701–711 (2005).
8. Camacho, J., Parrilla, M. & Fritsch, C. Phase coherence imaging. *IEEE Trans. Ultrason., Ferroelectr., Freq. Control* **56,** 958–974 (2009).
9. Camacho, J., Fritsch, C., Fernandez-Cruza, J. & Parrilla, M. *Phase Coherence Imaging: Principles, applications and current developments* in *Proceedings of Meetings on Acoustics ICU* **38** (2019), 055012.
10. Stratoudaki, T., Clark, M. & Wilcox, P. D. *Adapting the full matrix capture and the total focusing method to laser ultrasonics for remote non destructive testing* in *2017 IEEE International Ultrasonics Symposium (IUS)* (2017), 1–4.
11. Budyn, N., Bevan, R. L., Zhang, J., Croxford, A. J. & Wilcox, P. D. A model for multiview ultrasonic array inspection of small two-dimensional defects. *IEEE Trans. Ultrason., Ferro-electr., Freq. Control* **66,** 1129–1139 (2019).
